# Tabes Mesenterica

**Published:** 1879-01

**Authors:** S. W. Johnson

**Affiliations:** Atlanta, Ga.


### TABES MESENTERICA.

BY S. W. JOHNSON, M.D., ATLANTA, GA.

The lymph apparatus within the small intestines, both vessels and glands, is possessed of greater interest than is usually considered, as it takes part in nearly all affections of the intestinal canal. All the inflammatory affections of the canal may cause more or less swelling and reddening (hyperæmia) of the mesenteric glands ; they are, however, more particularly and seriously involved in typhoid fever, tuberculosis and scrofula.

The term *tabes mesenterica* is generally applied to any engorgement or tubercular degeneration of the mesenteric glands with emaciation and derangement of nutrition. More properly it is a scrofulous affection of the glands and is most frequently found in children.

The typhoid process usually causes no other changes than the medullary swelling, but still cases occur in which larger or smaller portions of the parenchyma become of a yellow color, or sometimes in a state of complete softening, those opposite the termination of the ileum in the colon being involved, the process extending from the intestine along the lacteals to the glands.

In tuberculous affection of the mesenteric glands the process also advances from the intestine towards the root of the mesentery ; it is always the row of glands nearest the intestines that first presents the tubercular eruption or greatest change.

The tubercles first appear in the cortical portion of the glands, but the medullary portion is not exempt, and as they undergo cheesy degeneration, the whole glandular parenchyma may become converted into a homogeneous, yellow, cheesy mass.

In scrofulous affections of these glands they also become cheesy and much enlarged—in children they become as large as pigeon's eggs and in adults still larger. The largest nodules are formed by a confluence of a number of the glands, and are frequently found at the root of the mesentery. They appear so yellow, firm and homogeneous upon section that the surface has been compared to the section of a raw potato, the moisture only being absent. The process does not, as in the other two conditions, advance from the intestine along the lacteals to the mesenteric glands, but like scrofula in other parts of the lymphatic system, it is first developed in the gland itself. In some instances the number of glands involved, or the enlargement of those diseased, is not sufficient to produce that amount of obstruction to the passage of chyle, which would interfere materially with nuitrition ; the chief source of danger, however, from this disease proper, is from the obstruction the chyle meets with at the cacoplastic gland, preventing its passage through them and into the thoracic duct.

The symptoms of the disease are not very positive or diagnostic. So long as the glands remain only moderately enlarged, buried as they are beneath the small intestines, it is difficult to detect their presence by palpation ; especially is this the case when the enlargement is confined mainly to the glands near the root of the mesentery. The most prominent symptoms, and those to be mainly relied upon, when the presence of the enlarged glands can not be detected, are : emaciation, progressing more rapidly or slowly, in proportion to the prevention of absorption of chyle ; anorexia, or a capricious appetite ; diarrhœa ; swelling of the abdomen, which is more of a globular than oval shape ; listlessness ; absence of any particular pain, and, towards the end, hectic fever. The prognosis is generally quite unfavorable, but we should bear in mind the possibility of recovery, where the hereditary tendency is not too strongly pronounced and the actual tuberculous deposit not excessive or rapidly progressing.

The treatment must, of course, be upon general principles, the nature of the cause and the stage of the disease being taken into account. If dependent upon a tuberculous diathesis, the remedies appropriate in tuberculous deposits in other portions of the system, as in the lungs, spleen and bronchial glands, are also appropriate in this affection. Where the enlargement of the glands is due to scrofulous cachexia, iodine in the form of the compound tincture, solution or iodide of potassium—iron and its various preparations, and cod liver oil, are indicated. The syrup of the iodide of iron appears to be especially useful, and this may be well given alternately or in conjunction with the iodide of potassium.

Where the cod liver oil can not be administered internally, we think good results may be obtained here, as under similar circumstances in other wasting diseases of children, by the use of the oil by inunction.

When the glands are enlarged to the extent of interfering materially with the passage of the chyle through them, we think attention to the diet of the child is of the very greatest importance ; the most bland, unirritating, digestible, and, at the same time, nourishing food being selected. Of course, strict attention should be given to dress and other hygienic measures. When the diarrhœa is excessive, the various astringents, in combination with opium, should be given freely.

If the enlargement be due to a previous acute or chronic inflammation of the intestinal canal, mercury, muriate of ammonia and counter-irritation by means of friction, dry cupping or pustulants, in conjunction with the proper regulation of diet are the proper measures to be adopted.

During the month of October last, I was called to see a negro child, three years old. It had been treated during the previous summer by a city physician of this place for typhoid fever, and had been in very feeble health ever since. Was very much emaciated—almost a "living skeleton" ; it lay with eyes half closed, in a listless mood, being perfectly indifferent to everything transpiring around it. Abdomen considerably enlarged ; occasional diarrhœa, alternating with constipation; appetite generally good ; no cough, lungs sound. The mother and father apparently in good health, both denying having ever had syphilis. They had two other children which presented no appearance of constitutional trouble.

We first suspected worms, and accordingly ordered an anthelmintic composed of santonin and calomel, which caused one small worm (lumbricus) to be passed the following day. On our second visit, two days from the first, we decided, after a more thorough examination—although unable to detect positively the enlarged glands—that it was a case of *tabes mesenterica* from scrofulous enlargement. It was too late, however, for remedies to benefit the child, as it died two days later.

Assisted by Drs. J. G. Westmoreland, J. O. Perkins, and Mr. C. C. Maddox, we performed a post mortem examination which verified our diagnosis. Most of the glands were enlarged and in a state of cheesy degeneration ; they were of all sizes, from that of a millet seed to a pigeon's egg—the largest being located near the root of the mesentery, nodules of the glands being found here as large as the child's fist. No lesion was to be found in the intestines or other abdominal viscera.

